# Gross Tumor Volume Predicts Survival and Pathological Complete Response of Locally Advanced Esophageal Cancer After Neoadjuvant Chemoradiotherapy

**DOI:** 10.3389/fonc.2022.898383

**Published:** 2022-06-07

**Authors:** Rong Wang, Xiaomei Zhou, Tongxin Liu, Shuimiao Lin, Yanxia Wang, Xiaogang Deng, Wei Wang

**Affiliations:** Department of Radiation Oncology, Nanfang Hospital, Southern Medical University, Guangzhou, China

**Keywords:** esophageal cancer (EC), neoadjuvant chemoradiotherapy, gross tumor volume (GTV), pathological complete response (PCR), survival analysis

## Abstract

**Background:**

Neoadjuvant chemoradiotherapy (neo-CRT) plus surgery has greatly improved the prognosis of locally advanced esophageal cancer (EC) patients. But which factors may influence the pathological tumor response and long-term survival remains unclear. The purpose of this study was to identify the prognostic biomarkers of locally advanced EC patients receiving neo-CRT.

**Methods:**

We reviewed the data of 72 patients with cT2-4N0-3M0 EC who underwent neo-CRT at our hospital. The patients received intensity-modulated radiation therapy with a total radiation dose of 41.4–60.0 Gy. Most patients received platinum + paclitaxel-based combination regimens every three weeks for 2–4 cycles. The recorded data included age, sex, smoking history, alcohol use, histology, tumor location, clinical TNM stage, tumor length, gross tumor volume (GTV), GTV of primary tumor (GTVp), GTV of lymph nodes (GTVn), radiation dose, and number of chemotherapy cycles. Overall survival (OS), progression-free survival (PFS), and pathological complete response (pCR) were analyzed.

**Results:**

The 3-year OS and PFS rates of these patients who underwent neo-CRT were 51.14% and 43.28%, respectively. In the univariate analyses, smoking history, clinical stage, GTV, GTVp, and GTVn were significantly associated with OS, whereas alcohol use, GTV, GTVp, and GTVn were significantly associated with PFS. Furthermore, in the multivariate analysis, GTV was an independent prognostic predictor of OS (hazard ratio (HR): 14.14, 95% confidence interval (CI): 3.747–53.33, *P* < 0.0001) and PFS (HR: 6.090, 95% CI: 2.398–15.47, *P* < 0.0001). In addition, GTV < 60.50 cm^3^ compared to > 60.50 cm^3^ was significantly associated with higher pCR rate (59.3% and 27.8%, respectively, *P* = 0.038). High dose (> 50 Gy) and increased number of chemotherapy cycles (≥ 3) didn’t improve the OS or PFS in patients with GTV > 60.50 cm^3^.

**Conclusion:**

GTV was an independent prognostic factor of long-term survival in EC patients, which may be because GTV is associated with histological response to neo-CRT. Additionally, patients with GTV > 60.50 cm^3^ didn’t benefit from increased radiation dose or increased number of chemotherapy cycles.

## Introduction

Esophageal cancer (EC) is the seventh most frequently diagnosed cancer and the sixth leading cause of cancer-related death worldwide (1). In particular, Asia has a high prevalence of EC, accounting for over 50% of the global morbidity and mortality (2); more than 90% of EC patients have esophageal squamous cell carcinoma. Based on the results of the CROSS (3) and NEOCRTEC5010 (4) studies, neoadjuvant chemoradiotherapy (neo-CRT) followed by surgery is recommended by the National Comprehensive Cancer Network, European Society for Medical Oncology, and Chinese Society of Clinical Oncology guidelines as the standard treatment modality for patients with non-metastatic thoracic EC (5, 6). Neo-CRT and surgery significantly improved the 5-year survival rate of EC patients compared to those undergoing surgery alone (7). However, the clinical application of neo-CRT has certain limitations. First, the clinical outcomes of neo-CRT vary between studies and the pathological complete response (pCR) rates range from 28% to 43.2% (3, 4, 8, 9). Second, compared to EC patients who underwent surgery alone, those receiving neo-CRT experienced more adverse events and might get disease progression due to delay in surgery. Therefore, it is imperative to identify patients who are likely to benefit from neo-CRT, to improve the efficacy of neo-CRT and establish appropriate treatment strategies.

The prognostic predictors of EC patients receiving neo-CRT are unclear. Previous studies have reported TNM stage (4, 10, 11), lymphatic invasion (12, 13), tumor grade (14), and age (15) as independent predictors of long-term survival of EC patients. However, EC patients with the same TNM stage may have different outcomes. Additionally, maximal esophageal wall thickness (16–18) and tumor length (19, 20) were reported to be associated with survival, suggesting that tumor burden may be a prognostic factor for EC patients. However, esophageal wall thickness and tumor length only provide one-dimensional information, which do not accurately reflect the tumor burden. In light of this, gross tumor volume (GTV) is easy to determine based on the target delineation system, provides information regarding tumor thickness and length, and may be an accurate prognostic factor for EC patients.

In this study, we collected data on the aforementioned factors, including GTV as a comprehensive tumor burden marker, to identify prognostic factors for survival in EC patients.

## Methods

### Patients

This single-center, retrospective study of the outcomes of EC after neoadjuvant therapy was approved by the Research Ethics Committee of Nanfang Hospital, Southern Medical University. Between January 2017 and October 2020, 481 EC patients received radiotherapy at our institution. We excluded 403 patients who did not receive neo-CRT and 6 patients without complete medical records. Thus, 72 patients with clinical stages of cT2-4N0-3M0 were enrolled in the study. All patients were aged ≥ 18 with histologically confirmed EC with no distant metastasis who received neoadjuvant chemoradiotherapy and had complete survival and treatment information. Patients with distant metastasis or death within 1 month after surgery were excluded.

We retrospectively collected the clinical characteristics of patients, including age, sex, smoking history, alcohol use, histological type, tumor location, TNM stage, tumor length, GTV, GTV of primary tumor (GTVp), GTV of lymph nodes (GTVn), radiation dose and number of chemotherapy cycles. Tumor location was determined by endoscopy. A tumor 15 to 20 cm away from the superior incisor was considered as cervical, whereas tumors 20 to 25 cm, 25 to 30 cm, and 30 to 40 cm were considered upper thoracic, middle thoracic, and lower thoracic, respectively. The stage of EC was determined based on the eighth edition of the American Joint Committee of Cancer TNM staging system for EC. Pathologic responses to neo-CRT were determined by two pathologists using the criteria developed by the American Joint Committee of Cancer and College of American Pathologists, which are defined as follows: grade 0 (complete response), no viable cancer cells; grade 1 (moderate response), single or small groups of cancer cells; grade 2 (minimal response), residual cancer outgrown by fibrosis; and grade 3 (poor response), minimal or no tumor kill, extensive residual cancer.

### Protocol of Neoadjuvant Chemoradiotherapy

All patients received external beam radiation, using intensity-modulated radiation therapy, which was delivered using megavoltage equipment with photon energies of 6–8 MV. Before radiotherapy, the patients underwent contrast-enhanced computed tomography (CT) simulation at 3-mm slice thickness in the supine position with immobilization for stereotactic treatment. We determined the GTVp using the borders of the increased esophageal wall thickness on CT scan, hypermetabolic lesions on 18F-fluorodeoxyglucose-positron emission tomography/computed tomography PET-CT (18F-FDG PET/CT), and the tumor location on endoscopy and endoscopic ultrasound. GTVn was defined by the enlarged regional lymph nodes, i.e., lymph nodes with short diameter ≥ 1 cm (paraesophageal or tracheoesophageal groove ≥ 5 mm) on CT or endoscopic ultrasound, or lymph nodes with high standardized uptake value (except for inflammatory lymph nodes) on 18F-FDG PET/CT. The GTV consisted of GTVp and GTVn. Then, the GTV, GTVp, and GTVn were calculated in cubic centimeters using the Varian Eclipse system. The clinical target volume (CTV) included a 3 cm craniocaudal and a 0.5–0.8 cm radial margin around the GTVp, and a 1-cm craniocaudal and a 0.5–0.8 cm radial margin around the GTVn, which included the area of subclinical involvement. The planning gross target volume (PGTV) was determined by including an area of 0.5 cm around the GTV in all directions for tumor motion and set-up variations. The planning clinical target volume (PCTV) was determined by including an area of 0.5 cm around the CTV in all directions. The prescription dose for the PCTV was 41.4–50 Gy at 1.8–2 Gy per fraction over 4–5 weeks. The prescription dose for the PGTV was 41.4–60 Gy at 1.8–2 Gy per fraction over 4–6 weeks. All plan were optimized such as D95 (DV is the absorbed dose in V% of the volume) ≥ the prescription dose and D1cc ≤ 115% of the prescription dose. The normal tissue-dose constraints included Dmax < 45 Gy for spinal cord, V30 < 45% for heart, V20 < 25% for lungs, Dmax < 45 Gy for intestines, and V30 < 30% for liver. During radiotherapy, chemotherapy was administered with either paclitaxel and platinum every three weeks, or fluoropyrimidine and platinum every four weeks for 2–4 cycles. The median time from the last day of neoadjuvant chemoradiotherapy to surgery is 42 days (range 21–91).

### Surgery

In the present study, an open or thoracoscopic transthoracic esophagectomy was performed in all patients. McKeown procedure, including a right-sided thoracotomy, laparotomy and cervical incision, was usually used for tumors in the middle and upper thoracic esophagus. Ivor Lewis procedure including a right-sided thoracotomy and laparotomy, or Sweet procedure including a left-sided thoracotomy was usually used for tumors in the lower thoracic esophagus. Lymph node dissections were performed according to the tumor location.

### Follow Up

Patients were regularly followed up in the outpatient clinic or using telephone interviews. Clinical evaluations included a CT scan of the neck-, thorax-, and abdomen, performed every 3 to 6 months. An endoscopic examination and bone scan were performed to detect recurrence and metastasis when necessary. The patients were followed up until death. Overall survival (OS) was defined as the interval from the date of neoadjuvant chemoradiotherapy to the date of cancer-related death or last follow-up. Progression-free survival (PFS) was calculated from the date of neoadjuvant chemoradiotherapy until disease progression or death. Patients who were still alive or lost to follow-up were treated as censored data for the analysis of survival rates.

### Statistical Analysis

The statistical analyses were performed using SPSS software (version 23.0; IBM Corp., Armonk, NY, USA) and GraphPad Prism 8.0 software (GraphPad, La Jolla, CA, USA). Categorical variables were presented as numbers and percentages, and groups were compared using the χ^2^ test. Furthermore, continuous variables were expressed as means and standard deviations, and means were compared using the Student’s *t* test. Time-dependent receiver operating characteristic curve analysis was used to identify the optimal cut-off values of GTV, GTVp, GTVn and tumor length for predicting the 1-year OS, as well as to compare their predictive capacity. The survival time distribution was evaluated using the Kaplan-Meier method, and the log-rank test was used for comparisons. A multivariate Cox proportional-hazard regression model was used to identify independent prognostic markers. A two-tailed *P* < 0.05 was considered statistically significant.

## Results

### Clinical Features and Treatment Information

**Figure 1:**
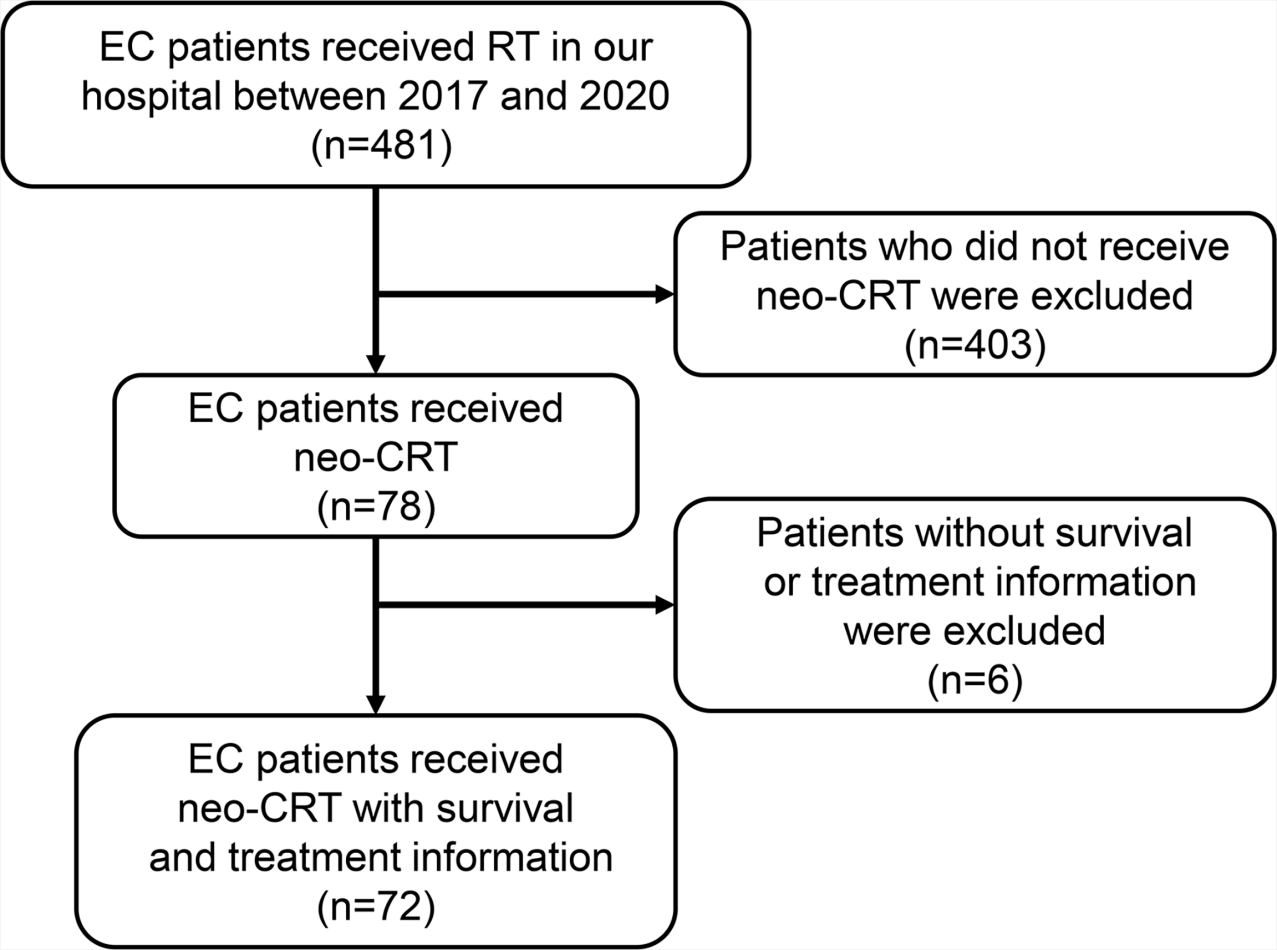
Flowchart of patient inclusion. EC, esophageal cancer; RT, radiotherapy; neo-CRT, neoadjuvant chemoradiotherapy.

To identify the prognostic factors for EC patients receiving neo-CRT, we reviewed the clinical information of 72 patients fulfilling the study’s eligibility criteria between 2017 and 2020 (**Figure 1**). The collected information included age, sex, smoking history, alcohol use, histology, tumor location, T stage, N stage, clinical stage, tumor length, GTV, GTVp, GTVn, radiation dose, and number of chemotherapy cycles. As shown in **Table 1**, a majority of patients were males (80.6%) with esophageal squamous cell carcinoma (95.8%), and nearly half had a history of smoking (59.7%) and alcohol use (48.6%). More than half of the cancers were located in the middle (44.4%) and lower (33.3%) esophagus, and the most common stages were T3 (76.4%) and N2 (40.3%). Most patients had locally advanced EC, i.e., stage III (56.9%) and IV (26.4%). In this study, concurrent chemoradiotherapy was used as neoadjuvant treatment. The most common radiotherapy dose for GTV was 50 Gy with a fractionated dose of 2 Gy (69.4%). Platinum + paclitaxel-based combination regimens were used in a large proportion of patients (88.9%), administered every three weeks for 2–4 cycles.

**Table 1:** Patients and treatment characteristics.

Variables	Study Cohort (n = 72) *
Age (yr)^†^	59.92 ± 7.99
Males	58 (80.6)
Smoking	43 (59.7)
Alcohol use	35 (48.6)
Histology	
SCC Others	69 (95.8) 3 (4.2)
Location	
Upper thoracic Middle thoracic Lower thoracic Others	15 (20.8) 32 (44.4) 24 (33.3) 1 (1.4)
T stage	
T2 T3 T4 Unknown	4 (5.5) 55 (76.4) 12 (16.7) 1 (1.4)
N stage	
N0 N1 N2 N3 Unknown	9 (12.5) 23 (31.9) 29 (40.3) 9 (12.5) 2 (2.8)
Clinical stage	
II III IVA Unknown	9 (12.5) 41 (56.9) 19 (26.4) 3 (4.2)
Tumor length (cm)^†^	6.57 ± 2.48
GTV (cm^3^)^†^	79.21 ± 70.08
GTVp (cm^3^)^†^	55.87 ± 35.92
GTVn (cm^3^)^†^	14.51 ± 17.95
Radiation dose	
> 50 Gy 50 Gy < 50 Gy	10 (13.9) 50 (69.4) 12 (16.7)
Chemotherapy regimen	
Paclitaxel + platinum Fluoropyrimidine + platinum	64 (88.9) 8 (11.1)
Chemotherapy cycles	
≥ 3 < 3	23 (31.9) 49 (68.1)

SCC, squamous cell carcinoma; GTV, gross tumor volume; GTVp, GTV of primary tumor; GTVn, GTV of lymph nodes.

*Except where indicated, data are numbers of patients (%).

^†^Data are mean ± standard deviation.

Additionally, we also summarized the information regarding tumor burden, including GTV (mean: 79.21 cm^3^), GTVp (mean: 55.87 cm^3^), GTVn (mean: 14.51 cm^3^), and tumor length (mean: 6.57cm). Using the receiver operating characteristic analysis method, we determined the optimal cut-off values to be 60.50 cm^3^, 41.45 cm^3^, 9.40 cm^3^ and 5.95 cm for GTV, GTVp, GTVn, and tumor length, respectively (**Supplementary Figure 1**). Then, patients were divided into two groups based on the optimal cut-off values for further analysis.

### Univariate and Multivariate Analyses of OS and PFS

**Figure 2:**
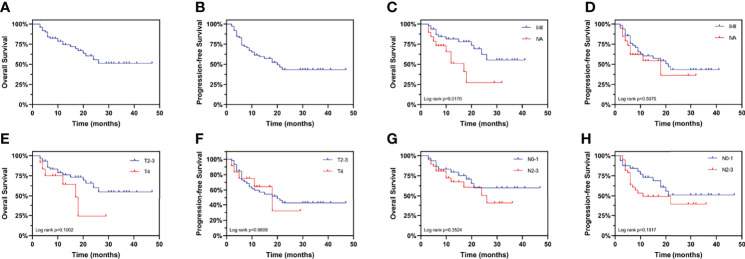
Kaplan-Meier curves for overall survival and progression-free survival stratified by clinical TNM stage. Curves are shown for overall survival in esophageal cancer patients **(A)** overall, **(C)** stratified by clinical stage, **(E)** stratified by clinical T stage, and **(G)** stratified by clinical N stage. Curves are shown for progression-free survival in esophageal cancer patients **(B)** overall, **(D)** stratified by clinical stage, **(F)** stratified by clinical T stage, and **(H)** stratified by clinical N stage.

All patients were followed up for a median period of 20 months (range 3-47). The 3-year OS rate was 51.14% (95% confidence interval (CI): 33.30–66.43%) (**Figure 2A**) and the 3-year PFS rate was 43.28% (95% CI: 28.85–56.88%) (**Figure 2B**), similar to the CROSS study, in which the 3-year OS rate of the chemoradiotherapy–surgery group was 58% (3). Currently, the TNM staging system is the most widely used tool for predicting the prognosis of EC patients. However, we found that the clinical stage was significantly associated with OS (**Figure 2C**), but not PFS (**Figure 2D**). In addition, neither the T stage (**Figures 2E, F**) nor the N stage (**Figures 2G, H**) was associated with OS or PFS, suggesting the need to identify other prognostic factors.

**Figure 3:**
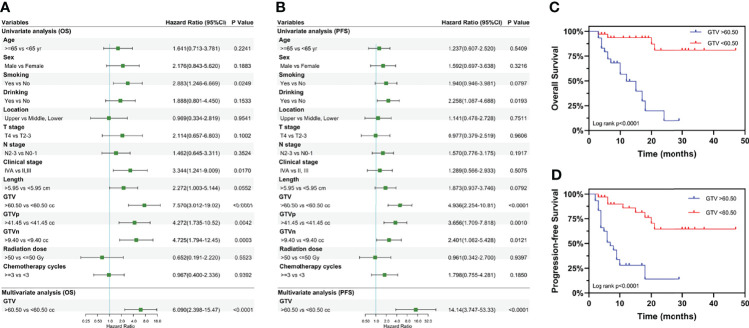
Univariate and multivariate analyses of overall survival and progression-free survival. Results of the univariate and multivariate analyses of the GTV effect on **(A)** overall survival and **(B)** progression-free survival. Kaplan-Meier curves for overall survival **(C)** and progression-free survival **(D)** stratified by GTV. Hazard ratios and 95% confidence intervals for death in the group with GTV > 60.50 cc, compared to the group with GTV < 60.50 cc. GTV, gross tumor volume; GTVp, gross tumor volume of primary; GTVn, gross tumor volume of lymph nodes; HR, hazard ratio; CI, confidence interval; cc, cubic centimeters.

The univariate analysis (**Figures 3A, B**) was performed using the abovementioned clinical features. Age, sex, location, T stage, N stage, tumor length, radiation dose and the number of chemotherapy cycles were not associated with OS or PFS. Smoking history (*P* = 0.0249) and clinical stage (*P* = 0.0170) were associated with OS, whereas alcohol use (*P* = 0.0193) was associated with PFS. Surprisingly, GTV, GTVp, and GTVn were significantly associated with both OS and PFS, with the largest survival difference for GTV. Multivariate Cox regression analysis showed that GTV was an independent prognostic factor for OS [hazard ratio (HR): 14.14, 95% CI: 3.747–53.33, *P* < 0.0001] and PFS (HR: 6.090, 95% CI: 2.398–15.47, *P* < 0.0001) in EC patients receiving neoadjuvant therapy (**Figures 3A, B**). Furthermore, as shown in **Figures 3C, D**, patients with GTV > 60.50 cm^3^ had shorter OS (HR: 7.570, 95% CI: 3.012–19.02, *P* < 0.0001) and PFS (HR: 4.936, 95% CI: 2.254–10.81, *P* < 0.0001) than those with GTV > 60.50 cm^3^.

### Prognostic Value of GTV

**Figure 4:**
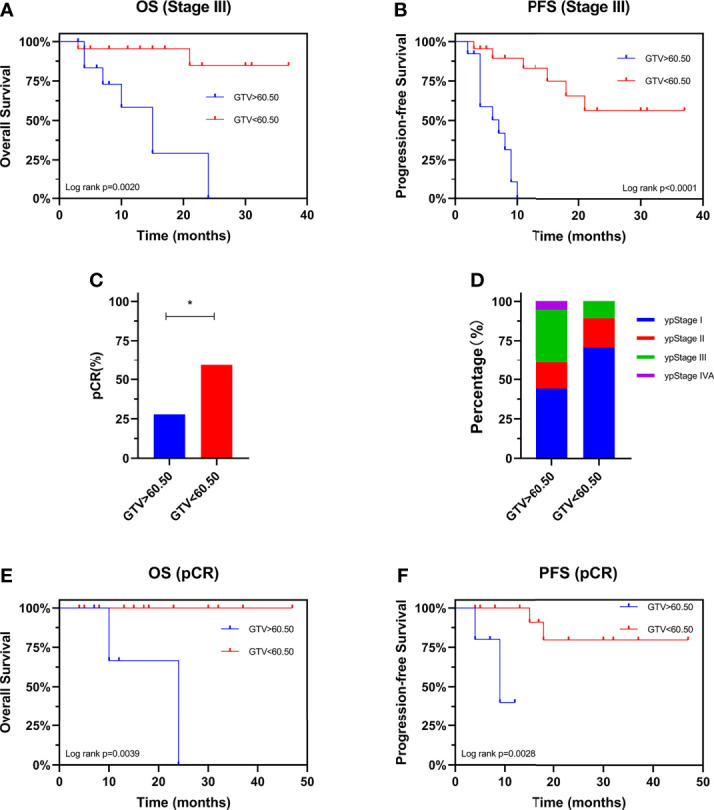
Prognostic value of GTV. Kaplan-Meier curves are shown for overall survival **(A)** and progression-free survival **(B)** stratified by GTV in patients with stage III disease. Pathological complete response rate **(C)** and ypStage **(D)** after neoadjuvant therapy stratified by GTV. Kaplan-Meier curves are shown for overall survival **(E)** and progression-free survival **(F)** stratified by GTV in patients achieving pCR. *P < 0.05 by χ2 test. GTV, gross tumor volume; pCR, pathological complete response; ypStage, post-neoadjuvant pathological stage.

**Figure 5:**
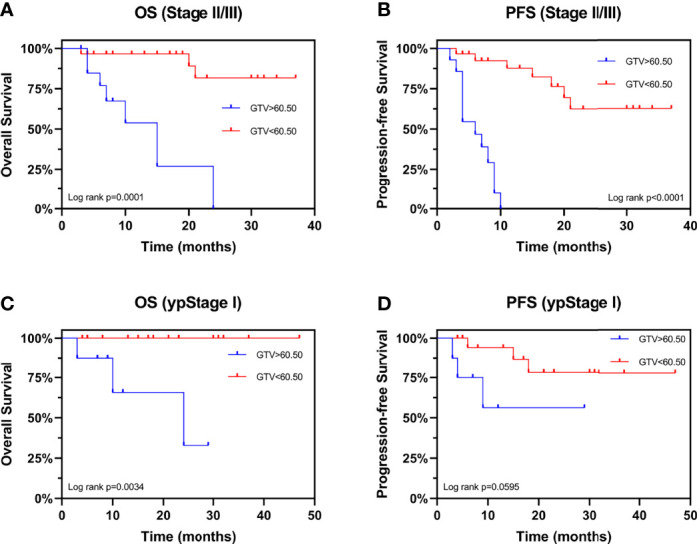
Kaplan-Meier Curves for overall survival and progression-free survival stratified by GTV. Curves are shown for overall survival stratified by GTV in patients with **(A)** stages II and III and **(C)** ypStage **I** Curves are shown for progression-free survival stratified by GTV in patients with **(B)** stages II and III and **(D)** ypStage **I.** GTV, gross tumor volume; ypStage, post-neoadjuvant pathological stage.

We evaluated the prognostic value of GTV in patients receiving neo-CRT with the same clinical stage. In stage III patients, GTV > 60.50 cm^3^ was associated with shorter OS (HR: 7.867, 95% CI: 1.670–37.07, *P* = 0.0020) and PFS (HR: 6.663, 95% CI: 2.098–21.16, *P* < 0.0001) (**Figures 4A, B**). These finding confirmed the independent prognostic value of GTV. To determine the basis of the relationship between GTV and prognosis, we explored the relationship between GTV and pCR rate after neo-CRT. The results showed that patients with GTV < 60.50 cm^3^ had higher pCR rate than those with GTV > 60.50 cm^3^ (59.3% and 27.8%, respectively, *P* = 0.038) and earlier post-neoadjuvant pathological stage after neoadjuvant therapy (**Figures 4C, D**). Moreover, even in patients achieving pCR (**Figures 4E, F**), GTV > 60.50 cm^3^ was associated with shorter OS and PFS. Similar results were found in patients with stages II and III (**Figures 5A, B**) or ypStage I (**Figures 5C, D**) after neoadjuvant treatment and surgery. Therefore, GTV is an important prognostic marker in EC patients.

### Survival Analysis Combining GTV and Treatment Information

**Figure 6:**
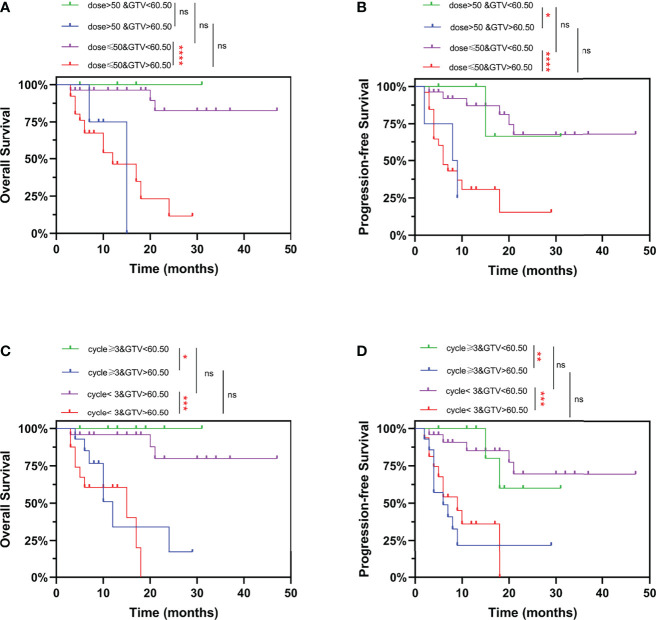
Kaplan-Meier curves for overall survival and progression-free survival stratified by GTV and radiation dose/number of chemotherapy cycles. Curves are shown for overall survival stratified by GTV and **(A)** prescription dose, or **(C)** number of chemotherapy cycles. Curves are shown for progression-free survival stratified by GTV and **(B)** prescription dose, or **(D)** number of chemotherapy cycles. ns, P > 0.05, *P < 0.05, **P < 0.01, ***P < 0.001, ****P < 0.0001 by log-rank test. GTV, gross tumor volume.

Finally, we combined GTV and treatment information for comprehensive analysis. Similarly, it was showed in **Figure 6** that patients with GTV > 60.50 cm^3^ had poorer OS and PFS than those with GTV < 60.50 cm^3^. Whereas, we found that increasing the radiation dose (**Figures 6A, B**) and the number of chemotherapy cycles (**Figures 6C, D**) did not improve OS and PFS, neither in EC patients with GTV > 60.50 cm^3^ nor < 60.50 cm^3^. In addition, for EC patients with GTV > 60.50 cm^3^, increased number of chemotherapy cycles (≥ 3) did not influence the pCR rate and downstaging rate after neo-CRT (**Supplementary Figure 2**). These results suggested that EC patients could not benefit from additional chemoradiotherapy. It is necessary to explore new treatment options to improve the prognosis of EC patients, especially those with GTV > 60.50 cm^3^.

## Discussion

The CROSS study suggested that neoadjuvant chemoradiotherapy is the preferred treatment for locally advanced EC patients (3). In the present study, an optimal cut-off GTV value of 60.50 cm^3^ was an independent prognostic factor for EC patients undergoing neo-CRT. Similar results were observed in patients with the same TNM stage, suggesting that the GTV may add valuable information to the TNM staging system. Furthermore, we found that patients with GTV < 60.50 cm^3^ had a better prognosis probably due to higher pCR rate. Patients with GTV > 60.50 cm^3^ did not benefit from increased radiation dose or increased number of chemotherapy cycles.

Pre-chemoradiotherapy maximal esophageal wall thickness on CT scan (odds ratio: 2.002, 95% CI: 1.075–3.728, *P* = 0.029) and tumor length (HR: 1.30, 95% CI: 1.21-1.40, *P* < 0.001) were independently associated with long-term survival (16, 21). Our results showed that large GTV correlated with poor OS (HR: 14.14, 95% CI: 3.747–53.33, *P* < 0.0001) and poor PFS (HR: 6.090, 95% CI: 2.398–15.47, *P* < 0.0001). GTV, as a three-dimensional factor, may be a better prognostic marker for EC patients than one-dimension factors. In the present study, the pCR rate of patients with GTV < 60.50 cm^3^ was 59.3%, which was significantly higher than the 49% in esophageal squamous cell carcinoma patients in the CROSS study (3). It has been reported that pCR after neo-CRT was associated with a better prognosis (22–24). Our results indicated that GTV may affect the prognosis by influencing the pCR rate.

The National Comprehensive Cancer Network guideline recommends a radiation dose of 41.4–50.4 Gy for neo-CRT, which remains controversial. Many studies have investigated the relationship between radiation dose and survival. Semenkovich et al. (25) suggested that high-dose radiation (> 50.4 Gy) did not improve tumor response, whereas Buckstein et al. (26) found no OS benefit to using doses > 41.4 Gy in neo-CRT for surgically resected EC patients. The ARTDECO study concluded absence of benefit to dose escalation in a phase III randomized setting, which is a topic of recent interest (27). Similarly, our study showed that the increase in radiation dose (> 50 Gy) and the number of chemotherapy cycles (≥ 3) may not improve the prognosis of patients with large GTV. EC patients could not benefit from additional chemoradiotherapy. Therefore, it is necessary to explore new treatment options, such as combinations with immune or targeted drugs, to improve the prognosis of EC patients, especially those with GTV > 60.50 cm^3^.

The present study revealed that large GTV leads to poor pCR rate and survival. This was one of the few studies to demonstrate the association between GTV and histological response to neo-CRT in locally advanced EC patients. However, limitations inherent in retrospective analyses also applied to our study. This was a retrospective study performed at a single institution; therefore, the results should be verified by prospective clinical studies. Makino et al. (28) reported that metabolic tumor volume change measured by 18F-FDG PET/CT before and after neoadjuvant chemotherapy predicted both long-term survival and histological response to preoperative chemotherapy in locally advanced EC patients. Therefore, tumor volume change may be a better marker for response to neo-CRT. Our study failed to explore the value of GTV change in predicting response to neo-CRT in EC patients because of the difficulty in determining GTV after radiotherapy. Finally, GTV could affect the prognosis of EC patients, but whether this was based on different biological backgrounds remains unclear. Further studies are needed to explore the biological mechanisms underlying the association of GTV and prognosis, which may provide new therapeutic targets for EC patients with large GTV.

## Conclusion

This study highlighted the important role of GTV in predicting long-term survival and histological response to neo-CRT. Patients with GTV > 60.50 cm^3^ did not benefit from increased radiation dose or increased number of chemotherapy cycles.

## Data Availability Statement

The original contributions presented in the study are included in the article/**Supplementary Material**. Further inquiries can be directed to the corresponding author.

## Ethics Statement

The studies involving human participants were reviewed and approved by the Research Ethics Committee of Nanfang Hospital, Southern Medical University. Written informed consent for patients was not required for this study in accordance with the national legislation and the institutional requirements.

## Author Contributions

All authors read and approved the final manuscript prior to submission. WW and RW are responsible for the conception and design of the study. RW and XZ are responsible for analysis and interpretation of data. XZ are responsible for drafting the article and revising it. TL, SL, YW, and XD are responsible for acquisition of data. All authors contributed to the article and approved the submitted version.

## Funding

This work was supported by the National Natural Science Foundation of China (grant number 82172642), the Outstanding Youths Development Scheme of Nanfang Hospital, Southern Medical University (grant number 2021J007), the GuangDong Basic and Applied Basic Research Foundation (grant number 2019A1515110931), and the President Foundation of Nanfang Hospital, Southern Medical University (grant number 2019L001).

## Conflict of Interest

The authors declare that the research was conducted in the absence of any commercial or financial relationships that could be construed as a potential conflict of interest.

## Publisher’s Note

All claims expressed in this article are solely those of the authors and do not necessarily represent those of their affiliated organizations, or those of the publisher, the editors and the reviewers. Any product that may be evaluated in this article, or claim that may be made by its manufacturer, is not guaranteed or endorsed by the publisher.

## Abbreviations

EC, esophageal cancer; neo-CRT, neoadjuvant chemoradiotherapy; GTV, gross tumor volume; GTVp, gross tumor volume of primary tumor; GTVn, gross tumor volume of lymph nodes; OS, overall survival; PFS, progression-free survival; pCR, pathological complete response; HR, hazard ratio; CI, confidence interval; CT, computed tomography; ^18^F-FDG PET/CT, ^18^F-fluorodeoxyglucose-positron emission tomography/computed tomography.
