# Supplementary material for: Gross Tumor Volume Predicts Survival and Pathological Complete Response of Locally Advanced Esophageal Cancer After Neoadjuvant Chemoradiotherapy
Source: Front Oncol. 2022 Jun 7;12:898383. doi: 10.3389/fonc.2022.898383 (PMC9209710; doi:10.3389/fonc.2022.898383)
Supplement: Supplementary file 1 [file DataSheet_1.docx]

Supplementary Material

# Supplementary Figures


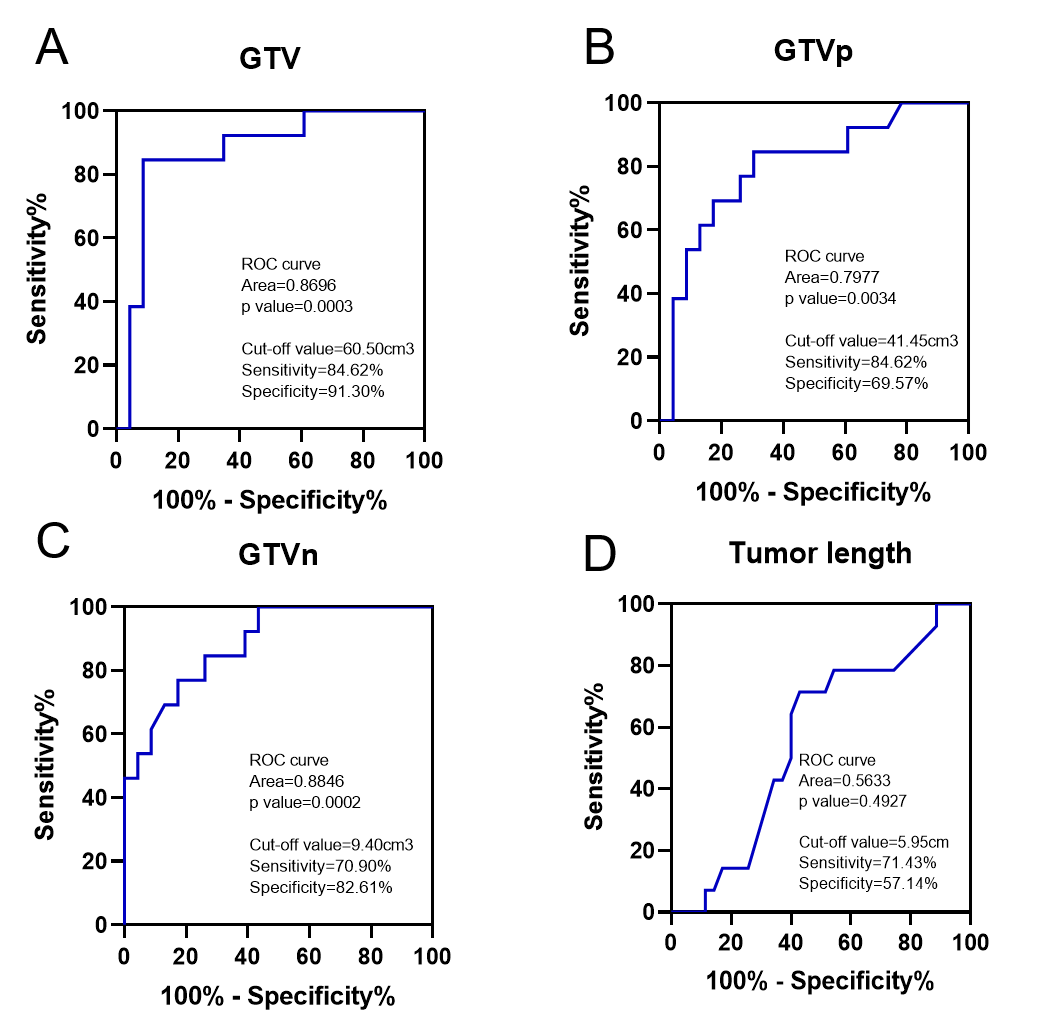


**Supplementary Figure 1.** **Receiver operating characteristics curve analyses.** The optimal cut-off values were identified for **(A)** GTV, **(B)** GTVp, **(C)** GTVn and **(D)** tumor length in patients receiving neoadjuvant therapy. GTV, gross tumor volume; GTVp, gross tumor volume of primary tumor; GTVn, gross tumor volume of lymph nodes.


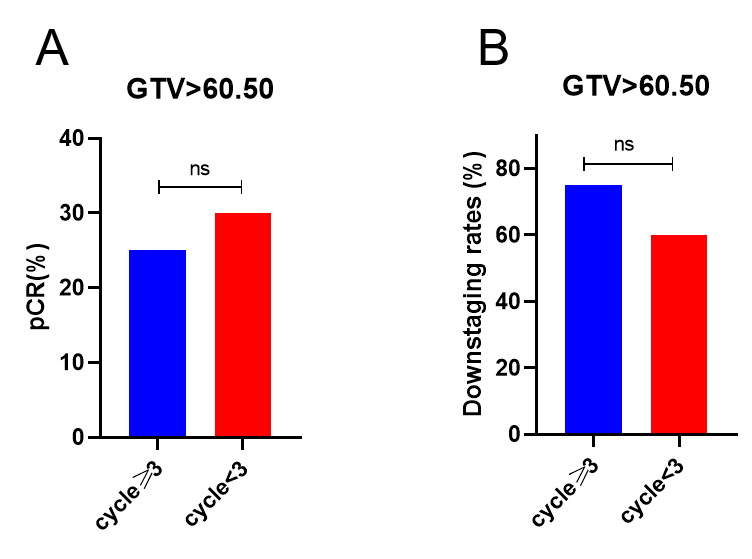


**Supplementary Figure 2. Pathological complete response rate (A) and downstaging rate (B) after neoadjuvant therapy stratified by number of chemotherapy cycles in patients with GTV > 60.50 cm^3^.** ns, P > 0.05 by χ^2^ test. GTV, gross tumor volume; pCR, pathological complete response.
